# Saphenous-sparing Ascending Video Endoscopic Inguinal Lymph Node Dissection Using a Leg Approach: Surgical Technique and Perioperative and Pathological Outcomes

**DOI:** 10.1016/j.euros.2021.10.004

**Published:** 2021-11-18

**Authors:** Christian D. Fankhauser, Esther W.C. Lee, Allaudin Issa, Pedro Oliveira, Maurice Lau, Vijay Sangar, Arie Parnham

**Affiliations:** a The Christie NHS Foundation Trusts, Manchester, UK; b Luzerner Kantonsspital, Lucerne, Switzerland; c University of Zurich, Zurich, Switzerland

**Keywords:** Penile cancer, Inguinal lymph node dissection, Video endoscopic inguinal lymph node dissection, Minimally invasive surgery

## Abstract

**Background:**

Open inguinal lymph node dissection (oILND) has high morbidity. Ascending saphenous-sparing video endoscopic ILND (VEILND-AS+) represents a minimally invasive alternative with potential benefits.

**Objective:**

To describe our VEILND-AS+ technique and compare outcomes to oILND.

**Design, setting, and participants:**

This was a retrospective cohort study of penile cancer patients.

**Surgical procedure:**

VEILND-AS+ was performed according to the technique described in the supplementary video.

**Measurements:**

We compared perioperative and pathological outcomes between the two procedures.

**Results and limitations:**

In the study cohort of 206 men we performed 40 VEILND-AS+ and 251 oILND procedures. In comparison to oILND, VEILND-AS+ had a longer operation time (185 vs 120 min; *p* < 0.01) but a shorter hospital stay (2 vs 4 d; *p* < 0.01). A median of eight resected lymph nodes with a median of one affected node per groin was observed in both groups. Extranodal extension was found in 30% of cases after VEILND-AS+ and 35% after oILND. In both groups the median drainage time was 13 d. Wound infections were observed in 38% of cases after VEILND-AS+ and 27% after oILND (*p* = 0.19). Skin necrosis or wound breakdown occurred in 0% and 6% of cases after VEILND-AS+ and oILND (*p* < 0.01), while lymphoceles were drained in 18% and 7% of cases, respectively(*p* = 0.03). Following VEILND-AS+ and oILND, 20% and 14% of patients, respectively, were referred to a lymph oedema clinic (*p* < 0.01).

**Conclusions:**

VEILND-AS+ is a safe procedure and offers shorter hospital stays and possibly a lower risk of skin necrosis and wound breakdown in comparison to oILND. Further improvements in the VEILND-AS+ technique are required to reduce complications associated with dead space and injury to lymphatic vessels.

**Patient summary:**

For patients undergoing surgery on lymph nodes in the groin, a minimally invasive approach instead of open surgery led to discharge 2 days earlier and may have lower rates of severe wound complications.

## 1 Introduction

Inguinal lymph node dissection (ILND) is an essential component in the management of penile cancer as it can assist in staging/prognostication and is therapeutic in men with lymph nodes metastases [1]. Open ILND (oILND) is accompanied by high morbidity [1] and therefore several surgical modifications have been suggested [2], [3], [4], [5], [6]. In this article we summarise our surgical technique and perioperative and oncological outcomes of ascending saphenous-sparing video endoscopic ILND (VEILND-AS+) and compare our results to our experience in a cohort of men undergoing oILND at a supraregional referral centre.

## 2 Patients and methods

### 2.1 Indication and preoperative staging

Men with histologically proven inguinal lymph node involvement according to either ultrasound-guided fine needle aspiration/core biopsy or dynamic sentinel lymph node biopsy (DSNB) using a gamma probe and blue dye are offered ILND. Before VEILND-AS+, men are staged using computed tomography of the chest, abdomen, and pelvis to assess sites of metastatic disease. In those with suspicion of femoral vessel involvement, oILND is performed. Men with fungating inguinal lymph nodes are referred for palliative radiotherapy/chemotherapy.

### 2.2 Informed consent

Before surgery, patients are counselled about their diagnosis, prognosis, and different treatment options, including oILND, VEILND-AS+, nonoperative care, and observation. The expected benefits, risks, and likelihood of success for each option are given and decision-making is augmented with procedure-specific information leaflets (www.christie.nhs.uk/patients-and-visitors/your-treatment-and-care/types-of-cancer/penile-cancer). Surgical complications are discussed, including nerve injury resulting in altered skin sensation or pain, infections/cellulitis, injury to vessels leading to bleeding/thrombosis, cancer recurrence, necrosis of inguinal skin, wound breakdown, inguinal hernia, injury to the spermatic cord leading to infertility and hypogonadism, seroma, lymphoedema (legs/scrotum/penis), deep vein thrombosis, and general complications of anaesthesia, and it is highlighted that any complication or disease recurrence may require further surgery.

### 2.3 Surgical procedure

#### 2.3.1 Patient and surgeon positioning and approach

The patient is positioned supine on the operating table in a frog leg position with a pillow below the knee. For unilateral VEILND-AS+, the hip is abducted and flexed to allow the knee to be flexed by 90° with the sole of the ipsilateral foot facing the contralateral knee supported by a sandbag to prevent movement. For bilateral VEILND-AS+, the hips are abducted and flexed to allow the knees to be flexed by 90° so that the legs mirror each other. The groin and thighs are shaved, prepared, and draped in a sterile manner so that the anatomical landmarks including the anterior superior iliac spine, inguinal ligament, pubic tubercle, sartorius muscle, and adductor magnus muscle are visible. A urinary Foley catheter is placed. The surgeon and assistant holding the camera are both positioned on the lateral side of the groin. The assistant is sitting in front holding the camera, while the surgeon is standing behind the assistant and the main monitor is close to the patient’s contralateral shoulder.

#### 2.3.2 Port placement and initial dissection

We first mark the resection boundaries of femoral triangle, which facilitates port placement. The cranial, caudal, lateral, and medial boundaries are the inguinal ligament, the apex of the femoral triangle, the sartorius muscle, and the adductor longus muscle [7]. The first skin incision of 3–4 cm is made 3 cm inferior to the apex of the femoral triangle with a scalpel blade. We prefer a 3–4-cm incision, which ensures good initial plane dissection and facilitates package retrieval. Using two Langenbeck retractors, the fascias of the adductor longus and sartorius muscles are displayed. More space for ports medially and laterally is developed initially using Langenbeck retractors for precise plane dissection, followed by blunt dissection with fingers following the already established planes. Care should be taken not to disrupt the delicate planes, and therefore the medial aspect between the muscles should preferentially be dissected under vision only. To avoid gas leakage, the first skin incision is reduced using a 1-0 polyglactin suture for a 12-mm Kii balloon port (C0R47; Applied Medical, Santa Margarita, CA, USA), which is blocked with 20 ml of air.

The gas pressure is initiated and maintained at 10 mm Hg. Using a 0° 10-mm laparoscope, the medial and lateral ports are positioned under vision. For right-handed surgeons we position a 12-mm AirSeal trocar (SurgiQuest, Milford, CT, USA) on the left side of the patient’s femoral triangle and a 5-mm Kii Fios balloon port (CFF03; Applied Medical) on the right side of the patient’s femoral triangle. Both the 5-mm and 10-mm ports are separated by a hand’s breadth from the visualising port to allow triangulation. As the AirSeal port initially has a tendency to slide into the port site and obstruct the camera view, we increase the resistance with several ties around the AirSeal shaft approximately 4 cm from the AirSeal tip.

#### 2.3.3 VEILND-AS+

The laparoscopic dissection is started on the fascia lata with a Johann grasper in the left hand and a dissection device (eg, THUNDERBEAT; Olympus, Tokyo, Japan) or a suction device for blunt dissection in the right hand. Care must be taken to correctly identify the muscle fascias, which are often visible as a “hang mat”. Dissection medially to the adductor longus muscles and laterally to the sartorius muscle is performed until the inguinal ligament can be visualised. The central part of the femoral triangle is dissected using a curved instrument (eg, right-angle or Maryland instrument) in the left hand and a dissection device or clip applier in the right hand. One or several long saphenous veins are identified normally originating from the medial aspect of the knee into the foramen ovale. The saphenous vein is dissected until the femoral vein is identified, and smaller tributaries are clipped or sealed with an energy device. Dissection cranial to the saphenous vein is continued until the inguinal ligament. The inguinal ligament is then cleared for 1–2 cm cranially from the pubic tubercle to the anterior superior iliac spine, exposing the spermatic cord as it exits the superficial inguinal ring.

The packet is now seen suspended superiorly in the operative view. The lymph node package is released by cutting upwards or “ascending” towards Scarpa’s fascia, identified as a white layer. Dissection then proceeds cranially, allowing the package to drop into the space created. The assistant or surgeon can palpate the depth of the skin flap over the instrument if necessary to ensure complete resection without impairing the vascular supply of the skin or even perforating the skin. In some men, the skin cranial to the inguinal crease is often angulating upwards and complete resection requires adjustment of the camera upwards and pulling of tissue downwards. After complete resection, the package is removed with a specimen retrieval bag. The resection plane is again inspected for meticulous haemostasis and flushed with sterile water.

#### 2.3.4 Drains, closure, and dressings

One 14 Fr suction drain is introduced through the lateral port and fixed twice to the skin and drain with 1-0 silk, as this drain may need to stay in place for several weeks. The camera port site is closed with a 2-0 polyglactin suture and all ports are closed with 3-0 Monocryl subcuticular sutures. The three incisions are covered with adhesive film dressings.

#### 2.3.5 Postoperative care

The patient can mobilise, eat, and drink immediately after surgery. Empirical antibiotic prophylaxis with amoxicillin clavulanate is prescribed for 3 d. Thromboprophylaxis with dalteparin sodium 5000 U is prescribed once daily for 28 d. On the first day after surgery, drains are cut and bagged, dressings are refreshed, and the patient is discharged with drains. District nurses are responsible for bag exchange and measurement of drain fluid volume. A clinical nurse specialist remains in contact with the patient on alternate days and organises drain removal as soon as the drain volume is <70 ml/24 h. No antibiotics are prescribed before drain removal.

#### 2.3.6 Common complications and management

In our experience, cellulitis resolves quickly after oral or intravenous amoxicillin clavulanate. Symptomatic lymphoceles are punctured and aspirated with or without ultrasound guidance. Any patient with lymphoedema is referred to the lymphoedema clinic. Wound breakdown is managed with packing or use of negative-pressure vacuum charcoal dressings.

### 2.4 Cohort study and video

The patients consented to the use of both photographic and videographic material for education in accordance with local institutional guidelines. Data were collected retrospectively for patients identified from surgical theatre diaries between 2005 and 2020. Perioperative and postoperative complications were extracted from medical charts by a board-certified urologist.

### 2.5 Statistical analysis

Descriptive statistics are used to report patient and tumour characteristics and perioperative and oncological outcomes. Operation time was defined from surgical sign-in to sign-out. Statistical analyses were performed using R version 3.1.3 (R Foundation for Statistical Computing, Vienna, Austria).

## 3 Results

Between 2005 and 2020, 206 men underwent ILND in 291 groins, with VEILND-AS+ implemented in 2018. The mean age was 63 yr and the median body mass index was 29 kg/m^2^; 13% of patients had a diagnosis of diabetes mellitus (Table 1). Bilateral oILND was performed in 71 patients, bilateral VEILND-AS+ in nine, oILND and VEILND-AS+ each on one side in five, unilateral oILND in 104, unilateral VEILND-AS+ in 16, and VEILND-AS+ and robotic ILND in one patient.

**Table 1:** Characteristics of 206 patients treated with inguinal lymph node dissection.

Parameter	Result
Mean age, yr (standard deviation)	63 (13)
Median body mass index, kg/m^2^ (interquartile range) [range]	29 (25–33) [19–47]
Diabetes, *n* (%)	27 (13)
T stage, *n* (%)	
T1a	16 (8)
T1b	26 (13)
T2	100 (49)
T3	48 (23)
T4	6 (3)
Missing	10 (4)
Grade, *n* (%)	
1	6 (3)
2	55 (27)
3	138 (67)
4	2 (1)
Missing	5 (2)
Histology, *n* (%)	
Squamous cell	176 (85)
Basaloid	20 (10)
Sarcomatoid	6 (3)
Missing	4 (2)
Local treatment, *n* (%)	
Biopsy only because of no primary tumour	3 (1)
Circumcision/wide local excision	35 (17)
Glansectomy	32 (16)
Partial penectomy	99 (48)
Total penectomy	37 (18)
Missing	0

For the per-groin analysis, the robotic LND was excluded, leaving 40 VEILND-AS+ and 251 oILND procedures available for analysis (Table 2). VEILND-AS+ compared to oILND required a longer operating time (185 vs 120 min; *p* < 0.01) but was associated with a shorter hospital stay (2 vs 4 d; *p* < 0.01). No VEILND-AS+ procedure was converted to oILND. In both the VEILND-AS+ and oILND groups, no patient returned to theatre for any reason. Pathological outcomes were similar, with a median of eight resected lymph nodes and a median of one affected node per groin in both groups. Extranodal extension was observed in 30% of VEILND-AS+ and 35% of oILND patients (*p* = 0.5).

**Table 2:** Outcomes after ILND stratified by surgical approach in per-groin analysis

Parameter	VEILND-AS+ (*n* = 40)	Open ILND (*n* = 251)	*p* value
**Perioperative data**			
Median operation time per groin, min (IQR)	185 (150–208)	120 (90–159)	<0.01
Median length of stay per admission, d (IQR)	2 (1–3)	4 (3–5)	<0.01
**Pathology data**			
Median number of nodes resected per groin, *n* (IQR)	8 (6.75–10)	8 (5–10)	0.56
Extranodal extension, *n* (%)	12/40 (30)	84/239 (35)	0.5
Not reported	0	12	
**Postoperative data**			
Median drainage time, d (IQR)	13 (8.5–27)	13 (5.5–20)	0.06
Skin-related complications, *n* (%)			
Cellulitis requiring antibiotic therapy	15/40 (38)	69/251 (27)	0.19
Skin necrosis or wound dehiscence	0/40 (0)	15/251 (6)	<0.01
Lymphatic complications, *n* (%)			
Lymphocele/seroma requiring drainage	7/40 (18)	18/251 (7)	0.03
Lymphoedema referred to lymphoedema clinic	8/40 (20)	14/100 (14)	<0.01

ILND = inguinal lymph node dissection; IQR = interquartile range; VEILND-AS+ = ascending saphenous-sparing video endoscopic ILND.

In the postoperative period, both groups showed the same median drainage time (13 d; *p* = 0.06). Wound infection requiring antibiotic therapy was observed in 38% of patients after VEILND-AS+ and 27% after oILND (*p* = 0.19). Skin necrosis or wound breakdown occurred in 0% and 6% of patients after VEILND-AS+ and oILND, respectively (*p* < 0.01), with lymphoceles or seromas drained in 18% and 7% (*p* = 0.03). Lymphoedema referrals were requested in 20% and 14% of cases after VEILND-AS+ and oILND, respectively (*p* < 0.01). Deep vein thrombosis and pulmonary embolism were observed in two men after bilateral oILND.

At median follow-up of 21 mo (interquartile range 8–54) after oILND, eight men experienced recurrence in the groin. At median follow-up of 12 mo (interquartile range 4–17) after VEILND-AS+, one man experienced recurrence in the groin.

## 4 Discussion

This report summarises our experience with VEILND-AS+ using a leg approach. Previous VEILND techniques described clipping [2] or stapling [3] of the saphenous vein despite saphenous sparing [4] being associated with a decrease in complications of 50% in a retrospective oILND cohort [5]. A saphenous-sparing VEILND technique was only described recently in a series of 23 patients without comparison to oILND and the technique was not described in a detailed step-by-step fashion [6]. Our data suggest that saphenous sparing during VEILND-AS+ is feasible and safe and associated with a short hospital stay and a lower risk of skin necrosis or wound breakdown. The lower rate of complications may not only improve quality of life and decrease overall treatment costs but may also decrease the time between groin surgery and subsequent pelvic lymph node dissection and/or adjuvant inguinopelvic radiotherapy. Furthermore, similar lymph node counts and proportions of men with extranodal extension indicate that VEILND-AS+ is as radical as oILND.

Our results suggest that VEILND-AS+ has a longer operation time and is not free of complications. The longer operation time may reflect limited experience in the first patients, representing a learning curve. The results for our cohort and others [8], [9], [10], [11] show comparable prolonged drainage time and symptomatic seroma or lymphoceles, suggesting that dead space and injury to lymphatic vessels is not resolved by VEILND-AS+ alone. In addition, two time-dependent developments in penile cancer care could explain our findings and represent potential confounders in favour of oILND. First, with increased knowledge regarding long-term consequences of untreated lymphoedema [12], early referral to lymphoedema services is now part of our pathway. Second, patients are often first seen in the community and have already started on empirical antibiotics before review by the operating team, which is a phenomenon more often observed since centralisation of care. In addition, recall bias by patients or more detailed clinical annotations by clinical nurse specialists with electronic health records could explain the greater proportions of men receiving empirical antibiotics.

Further surgical refinements are required to decrease the morbidity of ILND. The first promising surgical technique is prophylactic lymphovenous anastomosis and shunts [13], [14]. The second new approach worth exploring may include a lateral approach [15], [16], which could facilitate control of the distal medial lymphatics. A third development is hypogastric access [17], involving access to the pelvic and inguinal nodes through a single hypogastric route. Further modification involves use of a robotic system [18], [19], [20], [21] or single-port laparoscopic [22] or robotic system [23].

Given this study is a retrospective single-centre surgical review, it has significant limitations. Several small and single-arm cohorts suggest that further modifications may offer even lower morbidity. In the absence of comparison group selection, confounding is very likely because of substantial differences in indications and patient characteristics between centres. For example, an early VEILND cohort of ten Brazilian patients with penile cancer treated between 2003 and 2005 with a median lymph node count of ten experienced hardly any complications [24]. These early data suggest that ligation of distal lymphatic tissue at the femoral triangle vertex may result in lower complication rates and clipping of the saphenous vein may not increase complications. However, these early promising low complication rates may be explained by the fact that the only Brazilian men with nonpalpable lymph nodes without previous DSNB or biopsies were included, which is in contrast to our series of British individuals with a high proportion of palpable nodes and previous DSNBs. Furthermore, complications should be assessed prospectively [25] and definitions of symptomatic lymphocele/seromas, wound infections, and lymphoedema should be developed beforehand.

Given this limitation of previous results, we provide the first comparison of VEILND and oILND cohorts. However, such a comparison is prone to confounding and the sample size and low event rate do not allow adjustment for known confounders. Therefore, any new modification should ideally be evaluated in randomised controlled trials (RCTs) or at least prospective studies with a suitable comparator group. These studies must have well-defined inclusion criteria and endpoints, including definition of complications. In addition, the treatment protocol must be aligned across centres, as even among eUROGEN centres several important steps in the pathway differ substantially [7]. The present study has insufficient statistical power to comment on most outcomes, so our results should be regarded as hypothesis-generating and the primary goal of this report was to illustrate the surgical technique. Before a sufficiently powered RCT can be performed, we will randomise 50 patients to either oILND or VEILND across 12 institutions in the UK (NIHR200765). On the basis of this feasibility RCT and our retrospective data, we will then assess whether a sufficiently powered trial randomising patients to oILND versus VEILND is feasible.

## 5 Conclusions

VEILND-AS+ is a safe procedure compared to oILND and offers shorter hospital stays and possibly a lower risk of skin necrosis and wound breakdown. Further improvements in the VEILND-AS+ technique are required to reduce complications associated with dead space and injury to lymphatic vessels.

  ***Author contributions***: Christian D. Fankhauser had full access to all the data in the study and takes responsibility for the integrity of the data and the accuracy of the data analysis.

*Study concept and design*: Fankhauser, Issa, Parnham.

*Acquisition of data:* Fankhauser, Issa, Lee, Oliveira.

*Analysis and interpretation of data*: Fankhauser, Parnham.

*Drafting of the manuscript*: Fankhauser, Issa, Parnham.

*Critical revision of the manuscript for important intellectual content*: Oliveira, Lee, Lau, Sangar.

*Statistical analysis*: Fankhauser.

*Obtaining funding*: None.

*Administrative, technical, or material support*: None.

*Supervision*: Parnham, Sangar.

*Other*: None.

  ***Financial disclosures***: Christian D. Fankhauser certifies that all conflicts of interest, including specific financial interests and relationships and affiliations relevant to the subject matter or materials discussed in the manuscript (eg, employment/affiliation, grants or funding, consultancies, honoraria, stock ownership or options, expert testimony, royalties, or patents filed, received, or pending), are the following: None.

  ***Funding/Support and role of the sponsor***: None.

  ***Acknowledgments****:* We would like to thank Claire Phillips (Operating Department practitioner) and our clinical nurse specialists Jane Booker, Sharon Capper, Mandy Bell, Stephen Booth, Helen Johnson, and Catherine Pettersen for their excellent service to penile cancer patients.

## References

[1] Spiess P.E., Hernandez M.S., Pettaway C.A. (2008). Contemporary inguinal lymph node dissection: minimizing complications. World J Urol.

[2] Machado P., Stanczak M., Liu J.B. (2018). Subdermal ultrasound contrast agent injection for sentinel lymph node identification: an analysis of safety and contrast agent dose in healthy volunteers. J Ultrasound Med.

[3] Master V., Ogan K., Kooby D., Hsiao W., Delman K. (2009). Leg endoscopic groin lymphadenectomy (LEG procedure): step-by-step approach to a straightforward technique. Eur Urol.

[4] Catalona W.J. (1988). Modified inguinal lymphadenectomy for carcinoma of the penis with preservation of saphenous veins: technique and preliminary results. J Urol.

[5] Zhang S.H., Sood A.K., Sorosky J.I., Anderson B., Buller R.E. (2000). Preservation of the saphenous vein during inguinal lymphadenectomy decreases morbidity in patients with carcinoma of the vulva. Cancer.

[6] Cui Y., Chen H., Liu L. (2016). Saphenous vein sparing during laparoscopic bilateral inguinal lymphadenectomy for penile carcinoma patients. Int Urol Nephrol.

[7] Fankhauser C.D., Ayres B.E., Issa A. (2021). Practice patterns among penile cancer surgeons performing dynamic sentinel lymph node biopsy and radical inguinal lymph node dissection in men with penile cancer: a eUROGEN survey. Eur Urol Open Sci.

[8] Wang S., Du P., Tang X., An C., Zhang N., Yang Y. (2017). Comparison of efficiency of video endoscopy and open inguinal lymph node dissection. Anticancer Res.

[9] Kumar V., Sethia K.K. (2017). Prospective study comparing video-endoscopic radical inguinal lymph node dissection (VEILND) with open radical ILND (OILND) for penile cancer over an 8-year period. BJU Int.

[10] Schwentner C., Todenhöfer T., Seibold J. (2013). Endoscopic inguinofemoral lymphadenectomy—extended follow-up. J Endourol.

[11] Thyavihally Y.B., Dev P., Waigankar S.S. (2021). Comparative study of perioperative and survival outcomes after video endoscopic inguinal lymphadenectomy (VEIL) and open inguinal lymph node dissection (O-ILND) in the management of inguinal lymph nodes in carcinoma of the penis. J Robot Surg.

[12] Webb E., Neeman T., Bowden F.J., Gaida J., Mumford V., Bissett B. (2020). Compression therapy to prevent recurrent cellulitis of the leg. N Engl J Med.

[13] Boccardo F., Valenzano M., Costantini S. (2016). LYMPHA technique to prevent secondary lower limb lymphedema. Ann Surg Oncol.

[14] Jørgensen M.G., Toyserkani N.M., Sørensen J.A. (2018). The effect of prophylactic lymphovenous anastomosis and shunts for preventing cancer-related lymphedema: a systematic review and meta-analysis. Microsurgery.

[15] Jindal T., Meena M. (2021). Laparoscopic and robotic video endoscopic inguinal lymphadenectomy by the lateral approach. Asian J Endosc Surg.

[16] Elbalka S.S., Taha A., Srinivas C. (2020). Short-term surgical outcomes of standard and lateral video endoscopic inguinal lymphadenectomy: a multinational retrospective study. J Laparoendosc Adv Surg Tech A.

[17] Yuan P., Zhao C., Liu Z. (2018). Comparative study of video endoscopic inguinal lymphadenectomy through a hypogastric vs leg subcutaneous approach for penile cancer. J Endourol.

[18] Kharadjian T.B., Matin S.F., Pettaway C.A. (2014). Early experience of robotic-assisted inguinal lymphadenectomy: review of surgical outcomes relative to alternative approaches. Curr Urol Rep.

[19] Gkegkes I.D., Minis E.E., Iavazzo C. (2019). Robotic-assisted inguinal lymphadenectomy: a systematic review. J Robot Surg.

[20] Singh A., Jaipuria J., Goel A. (2018). Comparing outcomes of robotic and open inguinal lymph node dissection in patients with carcinoma of the penis. J Urol.

[21] Nabavizadeh R., Petrinec B., Nabavizadeh B., Singh A., Rawal S., Master V. (2021). Inguinal lymph node dissection in the era of minimally invasive surgical technology. Urol Oncol.

[22] Tobias-Machado M., Correa W.F., Reis L.O. (2011). Single-site video endoscopic inguinal lymphadenectomy: initial report. J Endourol.

[23] Fang A., Saidian A., Rosen J., Yarlagadda V., Nix J., Selph P. (2020). V02–11 Single-port robotic assisted laparoscopic bilateral inguinal lymph node dissection. J Urol.

[24] Tobias-Machado M., Tavares A., Ornellas A.A., Molina W.R., Juliano R.V., Wroclawski E.R. (2007). Video endoscopic inguinal lymphadenectomy: a new minimally invasive procedure for radical management of inguinal nodes in patients with penile squamous cell carcinoma. J Urol.

[25] Gandaglia G., Bravi C.A., Dell’Oglio P. (2018). The impact of implementation of the European Association of Urology Guidelines Panel recommendations on reporting and grading complications on perioperative outcomes after robot-assisted radical prostatectomy. Eur Urol.

